# Preliminary Results of National Amyotrophic Lateral Sclerosis (ALS) Registry Risk Factor Survey Data

**DOI:** 10.1371/journal.pone.0153683

**Published:** 2016-04-28

**Authors:** Leah Bryan, Wendy Kaye, Vinicius Antao, Paul Mehta, Oleg Muravov, D. Kevin Horton

**Affiliations:** 1 Carter Consulting Incorporated, Atlanta, GA, United States of America; 2 McKing Consulting Corporation, Atlanta, GA, United States of America; 3 Division of Toxicology and Human Health Sciences, Agency for Toxic Substances and Disease Registry, Atlanta, GA, United States of America; Institute for Health & the Environment, UNITED STATES

## Abstract

**Background:**

The National ALS Registry is made up of two components to capture amyotrophic lateral sclerosis (ALS) cases: national administrative databases (Medicare, Medicaid, Veterans Health Administration and Veterans Benefits Administration) and self-identified cases captured by the Registry’s web portal. This study describes self-reported characteristics of U.S. adults with ALS using the data collected by the National ALS Registry web portal risk factor surveys only from October 19, 2010 through December 31, 2013.

**Objective:**

To describe findings from the National ALS Registry’s web portal risk factor surveys.

**Measurements:**

The prevalence of select risk factors among adults with ALS was determined by calculating the frequencies of select risk factors—smoking and alcohol (non, current and former) histories, military service and occupational history, and family history of neurodegenerative diseases such as ALS, Alzheimer’s and/or Parkinson’s.

**Results:**

Nearly half of survey respondents were ever smokers compared with nearly 41% of adults nationally. Most respondents were ever drinkers which is comparable to national estimates. The majority were light drinkers. Nearly one-quarter of survey respondents were veterans compared with roughly 9% of US adults nationally. Most respondents were retired or disabled. The industries in which respondents were employed for the longest time were Professional and Scientific and Technical Services. When family history of neurodegenerative diseases in first degree relatives was evaluated against our comparison group, the rates of ALS were similar, but were higher for Parkinson’s disease, Alzheimer’s disease and any neurodegenerative diseases.

**Conclusions:**

The National ALS Registry web portal, to our knowledge, is the largest, most geographically diverse collection of risk factor data about adults living with ALS. Various characteristics were consistent with other published studies on ALS risk factors and will allow researchers to generate hypotheses for future research.

## Introduction

Amyotrophic lateral sclerosis (ALS) is a progressive and fatal neuromuscular disease. Most persons with ALS die within 2–5 years of becoming symptomatic [1]. Approximately 5–10% of ALS cases are estimated to be familial, of which over a dozen genes and loci of major effect have been identified [2]. The etiology of the remaining 90–95% of cases, commonly referred to as sporadic ALS, has challenged researchers since the disease was first described in 1869 by French neurologist Jean-Martin Charcot.

In October 2009, the United States (US) government launched its first and only population-based country-wide ALS registry as mandated by the National ALS Registry Act (S. 1382). This National ALS Registry allows researchers to quantify the incidence and prevalence of ALS in the United States, to describe the demographic characteristics of persons with ALS, and to examine risk factors for the disease. The Registry takes a two-pronged approach for tracking ALS cases in the United States by using 1) existing national administrative databases (i.e., Medicare, Medicaid, Veterans Heath Administration, and Veterans Benefit Administration) utilizing records starting from October 19, 2010 and 2) a secure web portal, launched on October 19, 2010, that allows patients to self-enroll [3]. During October 19, 2010–December 31, 2011, a total of 12,187 persons with ALS were identified by the National ALS Registry as living with the disease [4].

Most of what is known about ALS risk factors comes from epidemiological studies; however, the strength of evidence determined by these studies tends to vary. Being Caucasian (non-Hispanic), male, over 60 years, and having a family history of the disease are largely thought to be risk factors for ALS [1, 4–6]. Several studies have suggested an association between occupational exposures and ALS. The most common exposures investigated are pesticides or agricultural work [7–14], electromagnetic fields (EMF) [8, 15–20], metals (e.g., lead, selenium) [12, 14, 21–25], construction work [21], welding, soldering, and electric plating [24], formaldehyde [26], and diesel exhaust [27]. In addition, veterinarians, athletes, hairdressers, and armed forces personnel are professions that have been associated with ALS in an extensive systematic review [28]. Nutritional intake [29], exposure to infectious agents [30], cigarette smoking [31], and physical activity and trauma [32] also have been identified as possible risk factors. While epidemiological studies are necessary for attempting to determine ALS etiology, many studies contain a number of limitations for assessing the risk of developing the disease (e.g., small sample sizes, insufficient power, lack of representativeness, and limited geographic catchment areas).

Unlike previous studies of adults with ALS, the data utilized in this study are the largest collection of risk factor data about adults with ALS. This facilitated a more comprehensive examination of the characteristics among adults living with ALS than has been possible in past research. The primary objective of this paper is to describe the findings of risk factors including cigarette smoking, alcohol consumption, military service history, occupational history, and a family history of ALS completed via the National ALS Registry secure web portal.

## Methods

### Data Collection

Risk factor data were collected using a survey created and validated by the Stanford University School of Medicine’s ALS Consortium of Epidemiologic Studies (ACES) [33]. The survey was divided into shorter modules to facilitate completion over time, because of the possible physical limitations of the study population. Survey data were collected via a secure web portal from adults who have self-identified as having ALS. To verify ALS status, six questions proven to be reliable indicators for accurate ALS diagnoses in the U.S. Department of Veterans Affairs ALS registry were utilized [34]. Adults who satisfied the six validation questions were asked to provide consent by checking a box after reading the consent form, register online and participate in surveys. The study, including the consent form and consent procedures, was reviewed and approved by the Centers for Disease Control and Prevention Institutional Review Board (IRB).

### Survey Participation

There were 6,911 adults (age 18 or older) who registered via secure online portal between October 19, 2010 and December 31, 2013. To calculate survey participation rates, the number of adults who completed applicable surveys was divided by the number of registrants. Participation rates varied by survey module: demographics—54.0%; employment history—49.5%; military service history—48.6%; cigarette smoking and alcohol consumption—48.1%; first-degree relative history of ALS, Alzheimer’s disease or Parkinson’s disease diagnosis—46.0%.

### Measures

Survey questions used to define survey measures are in the Appendix. Race was collected using Office of Management and Budget (OMB) standard categories. Race was defined as the primary race if only one race was selected. If more than one race was chosen or race was Asian or Native Hawaiian or Pacific Islander, respondents were categorized as Other. If only ethnicity were specified or if respondent did not know race, race was defined as Unknown. Body mass index (BMI) was calculated using standard formula—BMI = weight (lb) / [height (in)]^2^ x 703. To determine change in BMI among survey participants, questions related to current and past BMI were asked. Baseline BMI was defined as the respondent’s BMI at 40 years old. Change in BMI was defined as the difference between current BMI and baseline BMI for respondents diagnosed after 40 years old. This measure was reported for two groups—adults over 40 years whose BMI had decreased or increased/remained the same. Geographic regions were created by combining US Department of Health and Human Services regions [35]. For these groupings, Northeastern region included HHS Regions 1,2,3; Southeastern region included HHS Region 4; Midwestern region included HHS Regions 5,7; Southwestern region included HHS Region 6; and Western region included HHS Regions 8,9,10. Exhaustive list of US states by region can be found in the Appendix. To determine occupational category, the North American Industry Classification System (NAICS), used by Federal statistical agencies in classifying business establishments for the purpose of collecting, analyzing, and publishing statistical data related to the U.S. business economy, was implemented. Self-reported family history of select neurodegenerative diseases was defined as having at least one first degree relative that has been diagnosed by a physician with ALS, Alzheimer’s disease and/or Parkinson’s disease. First degree relatives were defined as parent, sibling or child(ren). The occurrence of ALS, Alzheimer’s disease, Parkinson’s disease or any of those neurodegenerative diseases among siblings of adults with ALS was calculated by dividing the number of siblings with each condition by the total number of siblings. The rate among first degree relatives was calculated similarly for the total number of children, siblings and parents reported. Because a control group was not available for this study, the rates among the aforementioned groups were then compared to similar measures found during literature review [6]. Nationally representative estimates of risk factor prevalence among the U.S. general population are presented when applicable. SAS^©^ 9.3 was used to calculate the prevalence of risk factors among adults with ALS [36].

## Results

Demographic characteristics of web portal respondents are presented in Table 1. Most respondents reported being 50–69 years of age at registration. Among survey participants, 94% were white, 2% were black, 3% were Other and 60% were men. Most (96%) adults surveyed self-identified as non-Hispanic or non-Latino. Of survey respondents, 72% had completed some college or higher level of education. More than half of all survey responders reported currently living in the Southeast or Midwest regions of the United States. Among survey respondents who were older than 40 years and diagnosed after 40 years, half of respondents experienced a decrease in BMI when compared to their baseline BMI measure. More respondents diagnosed with ALS within two years but less than three experienced a decrease in BMI.

**Table 1:** Demographic Characteristics among Adults with ALS (October 19, 2010 –December 31, 2013).

	n	%
Age at Registration*		
18–39	276	4.0
40–49	943	13.6
50–59	1972	28.5
60–69	2328	33.7
70–79	1131	16.4
80+	258	3.7
Sex*		
Male	4137	59.9
Female	2774	40.1
Geographic Distribution*^,^^†^		
Northeast	1333	19.3
Southeast	1845	26.7
Midwest	1810	26.2
Southwest	591	8.6
West	1287	18.6
US Territories	13	0.2
Canada	3	0.0
**Race**^‡^		
White	3491	93.7
Black	87	2.3
Other	120	3.2
Unknown	29	0.8
**Ethnicity**^§^		
Hispanic or Latino	118	3.2
non-Hispanic or non-Latino	3588	96.3
**Education**		
Less than HS	111	3.0
HS graduate or GED	736	19.8
Technical or trade school diploma	215	5.8
Some college	718	19.3
College graduate	1170	31.4
Graduate degree	678	18.2
Other	98	2.6
**Change in Baseline BMI**^||^		
**Decreased BMI**	1499	50.2
ALS diagnosis less than 1 year ago	963	48.4
ALS diagnosis 1 or more years, but less than 2 years ago	232	53.5
ALS diagnosis 2 or more years, but less than 3 years ago	126	61.5
ALS diagnosis 3 or more years ago	178	50.3
**Equal/increased BMI**	1485	50.0
ALS diagnosis less than 1 year ago	1028	51.6
ALS diagnosis 1 or more years, but less than 2 years ago	202	46.5
ALS diagnosis 2 or more years, but less than 3 years ago	79	38.5
ALS diagnosis 3 or more years ago	176	49.7

All registrants (N = 6,911). Respondents with missing demographic information were excluded—(among all registrants) age at registration 3 (0.0%), state of residence 29 (0.4%); (among survey 1 respondents) ethnicity 21 (0.6%), education 1 (0.0%). Survey 1 (n = 3727).

* among all registrants,

^†^ Definition of geographic regions can be found in Appendix.

^‡^ Race was defined as either identifying with one racial group or, if racial identification included more than one racial group, respondents were classified as Other. If only Hispanic ethnicity and no racial group were chosen or if Race = Don't Know, race was defined as Unknown.

^§^ Ethnicity was defined as identifying with either Hispanic/Latino or non-Hispanic/non-Latino ethnic groups.

^||^ Change in baseline was calculated among those currently older than 40 years and diagnosed after 40 years. Time since diagnosis was calculated as difference between date of diagnosis and date of registration.

ALS risk behaviors are presented in Table 2. Among web portal survey respondents, rougly half were smokers. More men were ever smokers. Overall, of the 50% of ever smokers, more than 78% had smoked for more than 10 years and 76% smoked at least 5 pack-years. More men were ever drinkers. Overall, a total of 79% of respondents reported ever drinking; 86% drank at least one alcoholic beverage per month for more than 10 years. Approximately 69% were light drinkers as measured by the number of alcoholic drinks per month.

**Table 2:** Risk Behaviors among Adults with ALS (October 19, 2010 –December 31, 2013).

	Male		Female		Total	
Smoking Status*	n	%	n	%	n	%
Current Smoker	198	65.4	105	34.7	303	9.1
Former Smoker	862	64.1	483	35.9	1345	40.5
Nonsmoker	935	56.1	733	44.0	1668	50.2
**Ever Smoker**	1060	64.3	588	35.7	1648	49.6
**Smoking Duration**						
< 10 years	213	61.7	132	38.3	345	21.0
10- <25 years	414	65.2	221	34.8	635	38.5
25- <40 years	285	65.1	153	35.0	438	26.6
40+ years	144	65.5	76	34.6	220	13.4
**Smoking Intensity**^†^						
< 5 pack-years	214	57.7	157	42.3	371	22.5
5 to <15 pack-years	240	62.3	145	37.7	385	23.4
15+ pack-years	600	68.2	280	31.8	880	53.4
**Alcohol Consumption Status**						
Current Drinker	949	67.7	453	32.3	1402	42.2
Former Drinker	759	62.7	451	37.3	1210	36.4
Nondrinker	284	41.2	406	58.8	690	20.8
**Ever Drinker**	1708	65.4	904	34.6	2612	78.6
**Drinking Duration**						
< 10 years	184	51.7	172	48.3	356	13.6
10- <25 years	393	68.4	182	31.7	575	22.0
25- <40 years	684	64.8	372	35.2	1056	40.4
40+ years	437	71.6	173	28.4	610	23.4
**Drinking Intensity**^‡^						
Light Drinker	1048	58.5	743	41.5	1791	68.6
Moderate Drinker	320	78.8	86	21.2	406	15.6
Heavy Drinker	284	85.3	49	14.7	333	12.8

Missing smoking status 8 (0.2%), smoking duration 10 (0.6%), smoking intensity 12 (0.7%), alcohol consumption status 22 (0.7%), drinking duration 15 (0.6%), drinking intensity 82 (3.1%).

Survey 4 (n = 3324).

* Current smokers have smoked one or more cigarettes per day for six months or longer and still smoke; former smokers have smoked one or more cigarettes per day for six months or longer but currently do not; nonsmokers have not smoked one or more cigarettes per day for six months or longer. Current drinkers consumed alcoholic beverages at least once a month for 6 months or more and still drink; former drinkers consumed alcoholic beverages at least once a month for 6 months or more and do not currently drink; nondrinkers have not consumed alcoholic beverages at least once a month for 6 months or more.

^†^Pack-years are defined as multiplying the number of packs of cigarettes smoked per day by the number of years the person has smoked.

^‡^ Abel, EL, Kruger, ML, Friedl, J. (1998). How do physicians define "light", "moderate" and "heavy" drinking? 22(5) 979–984. Alcoholism: Clinical and Experimental Research.

History of military service is presented in Table 3. Veterans made up 24% of survey respondents with 35% serving in the Army, 23% serving in the Navy and 19% serving in the Air Force. Six percent of service members reported to have served in more than one branch of the military. Of those who have served, 34% have been deployed during times of military conflict. The military conflict with the largest proportion of deployed service members was Afghanistan (59%). Ten percent of those deployed were in Vietnam, and 10% of service members were deployed to more than one location.

**Table 3:** Military History among Adults with ALS (October 19, 2010 –December 31, 2013).

Military Service History		
	n	%
Veterans	789	23.5
Nonveterans	2561	76.2
**Branch of Military Service**		
Army	274	34.9
Navy	177	22.5
Marines	53	6.8
Air Force	150	19.1
Reserves	68	8.7
Coast Guard	17	2.2
More than one branch of military service	46	5.9
**Deployment to a War Arena**		
Yes	264	33.5
No	524	66.4
**Deployment Location**		
WWII	7	2.7
Korea	9	3.4
Vietnam	26	9.8
Persian Gulf	4	1.5
Afghanistan	156	59.1
Persian Gulf II	18	6.8
Other	20	7.6
More than one deployment location	24	9.1

Respondents with missing military service information—service 10 (0.3%), branch 4 (0.5%).

Survey 3 (n = 3360).

Occupational history is presented in Table 4. Over 33% of respondents were retired and nearly 39% were disabled at the time of registration. Jobs held for the longest time were Teacher, Professor or Educator (9%); Physician, Nurse, Dental or Health Care Worker (7%); Secretary, Administrative Assistant or Receptionist (5%); Engineer, Architect or Draftsperson (5%); Retail Salesperson, Sales Clerk, or Sales Representative (4%).Industries worked for the longest time were Professional, Scientific, and Technical Services (11%); Educational Services (11%); Health Care and Social Assistance (11%); Manufacturing (Metal, Electrical, Transport, Professional) (8%); Other Services (except Public Administration) (6%); Construction (6%); and Retail Trade I (Cars, Gas, Furniture, Electronics, Food-Beverage, Clothing) (6%).

**Table 4:** Occupational History of Adults with ALS (October 19, 2010 –December 31, 2013).

	n	%
**Employment Status (at Registration)**		
Full-time employed	603	17.6
Part-time employed	163	4.8
Retired	1122	32.8
Disabled	1318	38.5
Full-time student	5	0.2
Homemaker	63	1.8
Unemployed	87	2.5
Other	59	1.7
**Job Title Held for the Longest Time (Top 10)**		
Teacher, professor or educator	297	8.7
Physician, nurse, dental or health care worker	245	7.2
Secretary, administrative assistant or receptionist	168	4.9
Engineer, architect or draftsperson	157	4.6
Retail salesperson, sales clerk, or sales representative	146	4.3
Manufacturing laborer, production worker, or assembler/fabricator	118	3.4
Accountant, auditor, or bookkeeper	107	3.1
Supervisor or manager of financial or marketing workers	107	3.1
Chief executive or owner	91	2.7
Supervisor or manager of manufacturing or production workers	86	2.5
**Industry Worked in for the Longest Time (Top 10)**		
Professional, Scientific, and Technical Services	380	11.1
Educational Services	359	10.5
Health Care and Social Assistance	361	10.5
Manufacturing (Metal, Electrical, Transport, Professional)	265	7.7
Other Services (except Public Administration)	212	6.2
Construction	209	6.1
Retail Trade I (Cars, Gas, Furniture, Electronics, Food-Beverage, Clothing)	209	6.1
Finance and Insurance	183	5.3
Manufacturing—(Paper, Printing, Chemicals, Petroleum, Leather, Lumber, Stone)	138	4.0
Transportation and Warehousing I (Air, Rail, Water, Ground, Pipeline)	120	3.5
**Years of Employment at Longest Held Occupation**		
< = 10 years	497	14.5
10 < time < = 20 years	975	28.5
20 < time < = 30 years	1012	29.6
> 30 years	868	25.4

Respondents with missing employment history information were excluded—employment status 4 (0.1%), job title held for the longest time 57 (1.7%), industry worked in for the longest time 196 (5.7%), years of employment at longest held occupation 72 (2.1%). Survey 2 (n = 3424).

Family history is presented in Table 5. The rates of reported disease among siblings of adults living with ALS were lower for ALS, Parkinson’s disease, and all neurodegenerative diseases combined when compared with the rate of reported disease among siblings in the comparison group. However, the rate of disease among siblings was higher in our analysis (60 per 10,000) for Alzheimer’s disease than our comparison group (11 per 10,000). In our analyses, the rates of reported disease among all first degree relatives of adults living with Parkinson’s disease, Alzheimer’s disease, ALS, or any neurodegenerative disease was higher when compared with the rate of reported disease among all first degree relatives in the comparison groups.

**Table 5:** Family History of Adults with ALS (October 19, 2010 –December 31, 2013).

	Total # siblings	# siblings with condition	Rate of condition among siblings	Total 1st degree relatives*	# 1st degree relatives with condition*	Rate of condition among 1st degree*	# siblings with condition	Rate of condition among siblings	# 1st degree relatives with condition*	Rate of condition among 1st degree*	Ratio among siblings	Ratio among 1st degree*
ALS	8948	68	0.0076	21529	198	0.0092	13	0.0149	15	0.0084	0.5086	1.0950
Parkinson's Disease	9005	32	0.0036	21502	177	0.0082	4	0.0046	8	0.0045	0.7729	1.8377
Alzheimer's Disease	8903	53	0.0060	21460	467	0.0218	1	0.0011	12	0.0067	5.1792	3.2388
Neurodegenerative Disease	9005	153	0.0170	21529	842	0.0391	18	0.0207	35	0.0196	0.8212	1.9957

* 1st degree relative was defined as either parent(s), sibling(s) or child(ren).

Survey 6 (n = 3176).

## Discussion

Since October 19, 2010, over 35,000 risk factor surveys have been completed by Registry-enrolled persons with ALS. To our knowledge, this is the largest and most geographically diverse collection of risk factor data available about adults with ALS in the United States. Brief surveys about possible risk factors for ALS are available through the Registry’s secure web portal for completion by registrants. Findings from risk factor surveys may provide important insights into the pathology of ALS. Furthermore, the quantity of risk factor data collected helps to eliminate limitations traditionally associated with epidemiological studies (e.g., sample size, representativeness).

Date of registration was automatically captured by the National ALS Registry web portal and was, therefore, more reliable than a respondent-recalled diagnosis date which is subject to recall bias. The definition of diagnosis date may not be consistent if a respondent were to “self-diagnose” and recall the date of ALS symptom onset as his date of diagnosis versus the date that the ALS diagnosis was actually confirmed by a healthcare provider. This difference in time has been shown, on average, to be approximately 12 months[37]. As a result, registration date was used as the standard point of reference in this study. Because most risk factor surveys were completed at the time of registration, for consistency, survey respondent characteristics were described at the same point in time. The date of registration and date of diagnosis differed, on average, by less than two years which did not suggest a need to use date of diagnosis instead of date of registration.

Several studies have indicated that cigarette smoking may be related to the development of ALS [31, 38–41]. Smoking is thought to increase the risk of developing ALS through several mechanisms, including inflammation, oxidative stress and neurotoxicity caused by heavy metals and other chemical compounds present in cigarette smoke [22, 42, 43]. However, a definitive link between smoking and ALS has not been proven. Nationally, nearly 41% of adults have smoked in their lifetime[44]. In our study, half of adults were either current or former smokers. Of those ever smokers, more than half (52%) had 15 pack-years or more of smoking history. This level of tobacco exposure may be related to an increased risk of ALS [40, 41]. Additionally, the prevalence of ever smoking was somewhat higher than expected among women in our study (44%) when compared with women nationally (35%)[45] which is consistent with past research that suggests an increased susceptibility to ALS exists among women who smoke [40, 46]. The relationship between alcohol consumption and an increased risk of ALS is unclear. It is unknown whether alcohol consumption increases the risk of developing ALS or has a protective effect (38,40).

Occupational exposures such as military service have been linked with ALS [25, 47–50]. Veterans, regardless of branch of the military, deployed during times of combat were found to experience a higher risk of ALS compared to veterans who were not deployed during wartime [48] while another study links deployment to the first Persian Gulf War to ALS [47]. It remains unclear whether that increase is due to exposure to toxic or infectious agents resulting from deployment location or to some more general aspect of military life [47, 48]. Men who served in the military throughout the twentieth century might have been exposed to toxic agents (i.e., N,N-diethyl-m-toluamide, aerosolized lead (DEET, an insect repellant)) and various infectious agents [50]. One suggested hazardous exposure is the occupational exposure to diesel fuel of veterans from a variety of sources that generally occur during military conflict [51]. Nationally, 9% of adults are veterans [52]. In our analyses, veterans comprised 24% of survey respondents and about one-third (34%) of veteran respondents were deployed to at least one military conflict. Over one-half (56%) were deployed to the Afghanistan War and 9% in Persian Gulf Wars I and II combined. The overrepresentation of veterans who participated in the Afghanistan War may be explained by the age distribution of our study population who tended to be younger when compared to other ALS study populations rather than exposure to toxic or infectious agents specific to a deployment location.

As anticipated, given the incapacitating nature of ALS and the average age of diagnosis, more than three quarters of cases reported their employment status at registration as either disabled or retired. Among those reporting longest held occupation, teachers, health care workers, secretaries, salespersons, and engineers were the most common jobs. Although this might indicate that persons with higher education may be more likely to self-register, Gunnarsson et al. reported that office workers and medical service workers were more likely to have died from ALS in Sweden, with odds ratios of 1.8 (95% CI 1.0–3.3) and 1.7 (95% CI 1.0–3.0), respectively [11].

ALS patients also reported industries where they worked for the longest period. “Specialty trade contractors” and “Construction of buildings” appear among the top 10 of those industries. Both of these sub-categories belong to the construction sector, according to NAICS. Fang et al. conducted a case-control study in New England using 109 ALS patients and found an odds ratio of 2.9 (95% CI 1.2–7.2) for construction workers, excluding supervisors [21]. Construction workers may be exposed to many hazardous substances, depending on the specific task performed. These may include welding and electrical work, which have been associated with ALS. Strickland et al. demonstrated an odds ratio of 5.3 (95% CI 1.4–20.1) for workers exposed to welding or soldering materials [24]. Electric current injuries, also a common hazard among construction workers [53], have been associated with ALS in independent studies in the United States (odds ratio = 3.8 [95% CI 1.4–13.0]) [54] and Denmark (standardized mortality ratio = 2.7 [95% CI 1.0–6.0]) [55].

Studies have shown an increased risk of developing ALS among relatives who have a family history of ALS and other neurodegenerative diseases such as Parkinson’s disease and dementia [6, 56, 57]. This increased risk is reported for sporadic ALS cases as well as familiar ALS cases[6]. Previous studies have shown that persons with ALS have higher rates of neurodegenerative diseases among their family members [6, 56]. Because a control group was not available for this study, we compared the rates of disease in our population to those rates presented in the paper by Fallis [6]. Our results were very similar and showed a higher rate of neurodegenerative diseases reported in first degree relatives. It is unclear why the rate of disease in siblings was lower except for Alzheimer’s disease because Alzheimer’s disease was specifically asked and not dementia in general. Although the rates of disease were lower in siblings, they were still higher than the control group in this study. The Fallis study has limitations. Its study universe was a less populated area, Ireland, which may have resulted in smaller disease counts. However, similarities existed between our study and Fallis. While the comparison study does not provide racial data, it is assumed to be largely white because of its study location. The National ALS Registry population was also largely white. In addition, information about the same neurodegenerative diseases, Parkinson’s Disease, Alzheimer’s Disease and ALS was collected. The comparison study also limited its analysis to first-degree relatives, more specifically, siblings. Because of the scarcity of research about the familial occurrence of neurodegenerative diseases among ALS patients for specific types of first-degree relatives, additional research is necessary.

Previous studies have examined an association between an increased rate of BMI reduction and a shorter total disease duration [58, 59]. The distribution of the decrease in BMI relative to the time since ALS diagnosis in our study appeared to be consistent with past research. The decrease in BMI among longer term survivors, respondents who received their ALS diagnosis 3 or more years ago, was equivalent to that of respondents with an ALS diagnosis less than one year ago. This may indicate that respondents with a more rapid decrease in BMI, between one and less than three years, experienced a shorter disease duration that resulted in a survivor bias among adults who were diagnosed three or more years ago. These longer surviving respondents may have had other unique characteristics that facilitated their maintaining sufficient cognitive and physical ability that allowed registration and completion of risk factor surveys. This finding may suggest slower disease progression among this group. Further study is warranted.

There are several known limitations to this study. Our study population tended to differ demographically from the nation. Survey respondents were less racially diverse. This may be the result of ALS being more prevalent among whites and men [60, 61] or a reflection of racial disparity in Internet access and usage [62] which could result in a higher utilization of online ALS-related resources for particular groups. In addition, a majority of survey respondents were educated (74% had some college or higher) which has also been associated with higher Internet use [62]. However, of those who registered, there was no difference in age, sex, or geographic distribution between those who took surveys and those who did not take surveys. The underrepresentation of various demographic groups may result in an underestimation of the prevalence of risk factors among adults living with ALS. It is expected that more representative data will be collected as the Registry web portal matures. In addition, risk behavior surveys described in this study were completed by adults with ALS and not by a similar non-disease population. As a result, more definitive associations could not be tested. Thus, it was not possible to assess whether associations of risk factors of ALS were consistent or biased due to self-registration. However, general population national estimates were used as proxy comparison groups when applicable. Detailed information on specific exposures to hazardous agents and their duration and latency was not collected for occupational and military history risk factors, thereby, limiting the ability to extrapolate these results to a broader population or infer any causative links. Other potential exposures from such things as the use of home pesticides or hobbies were not assessed but have been added to information collected and will be analyzed in the future. Survey data were collected by self-reports which could underestimate negative health behaviors. However, past studies have shown some self-report measures to be sensitive and fairly specific [63, 64]. Answering surveys is voluntary and not everyone who registered took the surveys. In 2012, the Behavioral Risk Factor Surveillance System had a median response rate of 45.2% for all states and Washington, DC with a range from 27.7% - 60.4% [65]. Survey participation rates in the National ALS Registry ranged from 43.6%-49.2%, which is within the expected range for this type of research. Additionally, although every attempt was made to include only unique registrations in our analyses, there remains a possibility that duplicate registrations were included. However, further review of the data indicates that very few duplicate registrations likely existed, thus minimizing the effects on the overall conclusions. Results presented here include only National ALS Registry participants who completed risk factor surveys on the secure online web portal and may not be representative of all persons with ALS in the Registry. Lastly, these results were descriptive. Statistical testing including the control of potential covariates and confounders did not occur in this study. As a result, these finding should not be interpreted to suggest causation.

## Conclusions

These results encompass data on the largest number of persons with ALS in the United States to date. They were largely consistent with results of reported research on smaller less geographically diverse populations for cigarette smoking, alcohol consumption, military service history, occupational history and family history of neurodegenerative diseases. Our findings support the need for further investigation into the association between the development of ALS and a variety of risk factors, including, but not limited to, those presented in this paper. This risk factor data can be used to generate hypotheses to identify areas for future research and further examine the characteristics of adults with ALS.

## Appendix

**Table:** 

**Demographics**	
Height (post-baseline for respondents older than 40 years)	“What is your current height?”
Weight (post-baseline for respondents older than 40 years)	“What is your current weight?”
Height at 40 years old (baseline)	“What was your height at age 40 years?”
Weight at 40 years old (baseline)	“What was your weight at age 40 years?”
Race and/or ethnicity	Race was defined as the primary race. If more than one race was chosen or race was Asian or Native Hawaiian or Pacific Islander, respondents were categorized as Other. If only ethnicity were specified or if respondent did not know race, race was defined as Unknown. “Do you consider yourself Spanish, Hispanic, or Latino/Latina?” “What do you consider to be your race or ethnic group? If you belong to more than one of these groups, please indicate all groups that apply to you.”
**Risk Factors**	
Cigarette Smoking Status	“Have you ever smoked one or more cigarettes per day for six months or longer?”
Current Smokers	Respondents who smoked one or more cigarettes per day for ≥6 months and currently smoking.
Former Smokers	Respondents who smoked one or more cigarettes per day for ≥6 months but not currently smoking.
Nonsmokers	Respondents who had never smoked one or more cigarettes per day for ≥6 months.
Ever Smokers	Current or former smokers.
Smoking Duration	Ever smokers were asked, “During periods when you smoked, for how many years in total did you smoke?”
Smoking Intensity	Ever smokers were asked, “During periods when you smoked, how many cigarettes did you usually smoke in a day? One pack contains 20 cigarettes.” Cigarette smoking intensity was defined in pack years, the number of packs of cigarettes smoked per day per year of smoking.
Alcohol Consumption Status	“Did you ever drink alcoholic beverages such as wine, beer and spirits at least once a month for 6 months or more?”
Current Drinkers	Respondents who had consumed alcoholic beverages at least once a month for ≥6 months and currently drinking alcohol.
Former Drinkers	Respondents who had consumed alcoholic beverages at least once a month for ≥6 months and not currently drinking alcohol.
Nondrinkers	Respondents who had not consumed any alcoholic beverages in a month for ≥6 months.
Ever Drinkers	Current or former drinkers.
Drinking Duration	Ever drinkers were asked, "During periods when you were drinking alcoholic beverages, for how many years in total did you drink alcoholic beverages?” A drink is 12 oz. beer, 4 ounces of wine or a drink containing 1 oz. of liquor. Please select week or month.”
Drinking Intensity	Ever drinkers were asked, “During periods when you were drinking, how many alcoholic beverages did you usually have in a week OR month? A drink is 12 oz. beer, 4 ounces of wine or a drink containing 1 oz. of liquor. Please select week or month.” Light drinkers were defined as having consumed fewer than 8.4 alcohol drinks per week, moderate drinkers consumed between 8.4 and 15.4 alcohol drinks per week and heavy drinkers consumed more than 15.4 alcohol drinks per week(Abel 1998).
**Military Service History**	
Veterans Status	“Were you ever a member of the armed forces?”
Branch of Military Service	If yes, veterans were asked, “In which branch of service were you employed?” Available answer options were Army, Navy, Marines, Air Force, Reserves/National Guard or Coast Guard. Some veterans served in more than one branch of the military.
Deployment to War Arena	Veterans were asked, “Were you ever deployed to a war arena?” If yes, “To which war arena were you deployed?” Available answer options were World War II, Korean Conflict, Vietnam War, Persian Gulf, Afghanistan War, Persian Gulf II, Other. Some veterans were deployed to more than one deployment location. "
**Occupational History**	
Employment Status	“What is your current employment status?”
Job Title Held for the Longest Time	“Thinking about your entire working career, in which job were you employed for the longest period of time? Please indicate your job title and the industry in which you worked.”
Years of Employment at Longest Held Occupation	“For how many years were you employed in this occupation?”
Titles of longest held jobs and corresponding industries	predetermined drop-down menus
**Family History**	“Has your mother/father/brother/sister/child(ren) ever been diagnosed by a physician with any of these conditions—ALS, Alzheimer’s Disease, Parkinson’s Disease?
**Geographic Distribution**	
Northeastern region	HHS Regions 1,2,3
Southeastern region	HHS Region 4
Midwestern region	HHS Regions 5,7
Southwestern region	HHS Region 6
Western region	HHS Regions 8,9,10
Region 1—Connecticut, Maine, Massachusetts, New Hampshire, Rhode Island, Vermont	
Region 2—New Jersey, New York, Puerto Rico, the Virgin Islands	
Region 3—Delaware, District of Columbia, Maryland, Pennsylvania, Virginia, West Virginia	
Region 4—Alabama, Florida, Georgia, Kentucky, Mississippi, North Carolina, South Carolina, Tennessee	
Region 5—Illinois, Indiana, Michigan, Minnesota, Ohio, Wisconsin	
Region 6—Arkansas, Louisiana, New Mexico, Oklahoma, Texas	
Region 7—Iowa, Kansas, Missouri, Nebraska	
Region 8—Colorado, Montana, North Dakota, South Dakota, Utah, Wyoming	
Region 9—Arizona, California, Hawaii, Nevada, American Samoa, Commonwealth of the Northern Mariana Islands, Federated States of Micronesia, Guam, Marshall Islands, Republic of Palau	
